# Direction-of-Arrival Estimation Method Based on Neural Network with Temporal Structure for Underwater Acoustic Vector Sensor Array

**DOI:** 10.3390/s23104919

**Published:** 2023-05-19

**Authors:** Yangyang Xie, Biao Wang

**Affiliations:** School of Naval Architecture and Ocean Engineering, Jiangsu University of Science and Technology, Zhenjiang 212100, China

**Keywords:** underwater acoustic vector sensor array, signal processing, DOA estimation, long and short memory network, transformer

## Abstract

Acoustic vector sensor (AVS) is a kind of sensor widely used in underwater detection. Traditional methods use the covariance matrix of the received signal to estimate the direction-of-arrival (DOA), which not only loses the timing structure of the signal but also has the problem of weak anti-noise ability. Therefore, this paper proposes two DOA estimation methods for underwater AVS arrays, one based on a long short-term memory network and attention mechanism (LSTM-ATT), and the other based on Transformer. These two methods can capture the contextual information of sequence signals and extract features with important semantic information. The simulation results show that the two proposed methods perform much better than the multiple signal classification (MUSIC) method, especially in the case of low signal-to-noise ratio (SNR), the DOA estimation accuracy has been greatly improved. The accuracy of the DOA estimation method based on Transformer is comparable to that of the DOA estimation method based on LSTM-ATT, but the computational efficiency is obviously better than that of the DOA estimation method based on LSTM-ATT. Therefore, the DOA estimation method based on Transformer proposed in this paper can provide a reference for fast and effective DOA estimation under low SNR.

## 1. Introduction

Direction-of-arrival (DOA), as one of the important applications in the field of array signal processing, has always been the research frontier and hotspot in the field of signal processing. With the development of science and technology, the rapid increase of human underwater activities has led to the rise of underwater noise levels. At the same time, the manufacturing process of submarines and surface ships has improved, which weakens the target signal strength and further reduces the underwater signal-to-noise ratio (SNR) [1]. How to estimate the DOA quickly and efficiently under the condition of low SNR is the core task of underwater array signal processing. With the improvement of underwater target positioning requirements and underwater research equipment, acoustic vector sensor (AVS) has gradually come into the public’s view, providing a more solid foundation for accurate underwater target positioning. Compared with the traditional DOA estimation method based on acoustic pressure sensor array, the DOA estimation method based on AVS array is more diverse and has better performance. It has become one of the research hotspots in the field of array signal processing and marine positioning and recognition [2,3,4].

The main research task of DOA estimation is how to estimate the DOA of communication signals efficiently and quickly so that the DOA algorithm has real-time estimation ability and anti-noise ability in large array environments. Some traditional DOA estimation methods have some shortcomings. The traditional beamforming algorithm is affected by large side-lobe fluctuations [5]. While the MUSIC method has a strong dependence on the estimation accuracy of the array output covariance matrix and noise subspace, its performance seriously degrades in the case of low SNR and an insufficient number of snapshots [6,7,8]. Although many improved algorithms can alleviate the problem to some extent and make it more robust, they cannot eliminate the limitations caused by subspace decomposition in essence.

In recent years, DOA estimation methods based on deep learning [9,10,11,12,13,14,15,16,17,18,19,20,21] have become another research hotspot in the direction-finding field. This kind of method does not need to build a parameter model, but directly learns the nonlinear relationship between the array output and the incoming wave direction from the data set, so as to realize DOA estimation. Therefore, it is also called a data-based method. At the same time, although the DOA estimation method based on deep learning takes a lot of time in the offline training process, once the network training is completed, it is almost real-time to use it for DOA estimation. To further improve the estimation performance of DOA estimation methods based on deep learning, researchers mainly focus on constructing suitable feature extraction methods and designing reasonable network structures. At present, most DOA estimation methods based on deep learning take the array output covariance matrix and its variants as feature extraction methods. For example, the upper right matrix of the array output covariance matrix (excluding diagonal elements) is reconstructed into a vector form as the input feature [10]. With the diagonal elements unchanged, the real part of the upper triangle and the imaginary part of the lower triangle of the covariance matrix are taken to form the characteristic matrix [15]. The phase feature of the covariance matrix is taken as the input of the network [16]. In terms of network structure design, the existing DOA estimation methods based on deep learning adopt various neural network structures [10,12,13,14,15,16,17]. Among them, the convolutional neural network can greatly reduce the number of network parameters and simplify the network structure on the premise of ensuring the learning performance of the network due to its local receptive field and weight-sharing characteristics. Therefore, most DOA estimation methods based on deep learning adopt the convolutional neural network structure [12,14,15,16,17]. The main distinction between neural network methods and conventional methods lies in their underlying principles and approaches. The proposed methods learn nonlinear relationships between inputs and outputs using large amounts of training data, enabling them to capture complex patterns and improve estimation accuracy. On the other hand, conventional methods such as MUSIC rely on the decomposition of the covariance matrix and the separation of signal and noise subspaces. Meanwhile, the training phase in the neural network methods involves optimizing the network parameters based on a specific objective. This process is not directly compatible with the calculations involved in the MUSIC algorithm, such as covariance matrix estimation and eigenvalue decomposition.

Most of the above DOA estimation methods based on deep learning are about acoustic pressure sensor, while deep learning for AVS arrays is rarely applied. At the same time, the covariance matrix used in the above method is for the calculation of the mathematical expectation, which loses the time-series structure of the data and leads to the loss of some information. Therefore, this paper proposes a DOA estimation method that combines long short-term memory (LSTM) and attention mechanism (LSTM-ATT) firstly for AVS arrays. This method directly processes the time-series signal data and retains the information in the original signal as much as possible. However, this method takes a long time to model because the sequence structure can only run serially, and a single attention mechanism may have the risk of model overfitting. On this basis, in order to improve the calculation efficiency and estimation accuracy of this DOA estimation method, a DOA estimation method based on Transformer is established. Transformer divides the time-series signal data into multiple segments and sends them to multiple subspaces. Then, the self-attention mechanism in each subspace calculates the influence and, finally, combines to calculate the attention weight. Experimental results show that the DOA estimation method based on LSTM-ATT and the DOA estimation method based on Transformer are superior to the MUSIC method in accuracy and RMSE, and the computational efficiency of the DOA estimation method based on Transformer is greatly improved compared with the DOA estimation method based on LSTM-ATT.

## 2. Basic Principle of Acoustic Vector Sensor Array

### 2.1. Array Receiving Model

**Figure 1:**
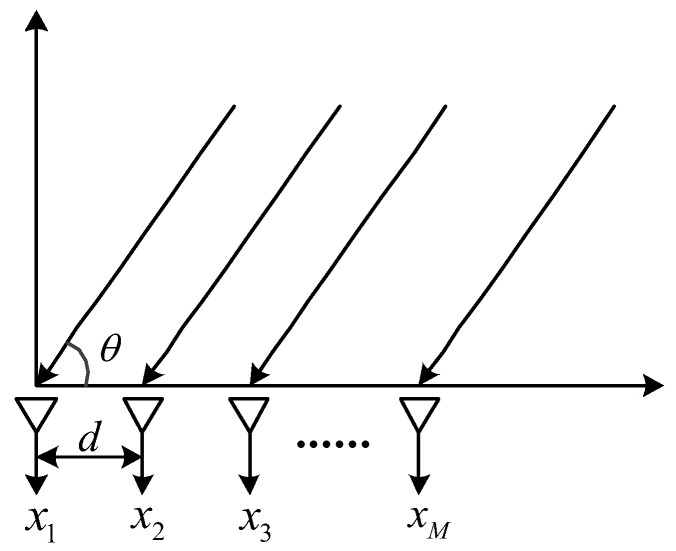
Model of the AVS array.

As shown in Figure 1, a uniform linear array composed of *M*-ary two-dimensional vector sensors is considered, and the distance between adjacent elements is half the wavelength d=λ/2. It is assumed that the received signal meets the narrowband condition, and the signal is a far-field signal. For a two-dimensional underwater AVS, if the angle of the vibration velocity direction relative to the x-axis is defined as *θ*, the output of a single vector sensor can be expressed as follows:

(1) $$x\left(t\right)=\left[\begin{array}{c} p\left(t\right)\\ v_{x}\left(t\right)\\ v_{y}\left(t\right) \end{array}\right]=\left[\begin{array}{c} s\left(t\right)\\ s\left(t\right)\cos\theta \\ s\left(t\right)\sin\theta \end{array}\right],$$

where s(t) represents the time domain signal of the incident wave. The unit amplitude response of a single AVS array element can be defined as follows:

(2) $$h(\theta )={\left[1\;\cos\theta \;\sin\theta \right]}^{T},$$

where ${\left[\cdot \right]}^{T}$ represents the transposition of the matrix. According to the wave path difference, the spatial phase delay vector expression of the linear AVS array can be obtained as follows:

(3) $$a_{p}(\theta )={\left[1,e^{j(2\pi /\lambda )d\cos(\theta )},e^{j2(2\pi /\lambda )d\cos(\theta )},\cdots ,e^{j(M-1)(2\pi /\lambda )d\cos(\theta )}\right]}^{T}$$

Then, the AVS array guidance vector is as follows:

(4) $$a(\theta )=a_{p}(\theta )⊗h(\theta ),$$

where ⊗ is expressed as a Kronecker product. Under *J* snapshots, the output data of this far-field narrowband signal incident on the uniform linear AVS array is as follows:

(5) $$X\left(t\right)=\left[\begin{array}{c} x_{1}\left(t\right)\\ \vdots \\ x_{M}\left(t\right) \end{array}\right]=A\left(\theta \right)S\left(t\right)+N\left(t\right)\;\;\;\;\;\;\;t=t_{1},\cdots ,t_{J},$$

where $x_{m}\left(t\right),m=1,\cdots ,M$ represents the time-domain output of the *m*-th array element, and $X\left(t\right)\in C^{3M\times J}$ represents the received data of 3*M* channels of the AVS array; $S\left(t\right)\in C^{1\times J}$ represents the time domain sampling of one group of far-field narrowband incident plane waves; $A\left(\theta \right)=a\left(\theta \right)\in C^{3M\times 1}$ is called the space flow pattern matrix of the vector matrix; and $N\left(t\right)\in C^{3M\times J}$ indicates the additive noise received by each channel.

### 2.2. Array Data Analysis and Processing

Because sound wave contains two parts of information, amplitude and phase angle, the received data obtained by the AVS array have complex values. Because complex data cannot be directly processed in the training process of a deep learning network, it is necessary to carry out corresponding mathematical processing on the received data. For the output data of the AVS array, X(t) can be divided into two parts, the real part and the imaginary part, so that it is as follows:

(6) {Q=Re{X(t)}P=Im{X(t)},

where Re{·} represents the real part of the complex number, and Im{·} represents the imaginary part of the complex number. Two independent real-valued matrices, *Q* and *P*, are used as the input data of the neural network.

## 3. DOA Estimation Method Based on Time Series

**Figure 2:**
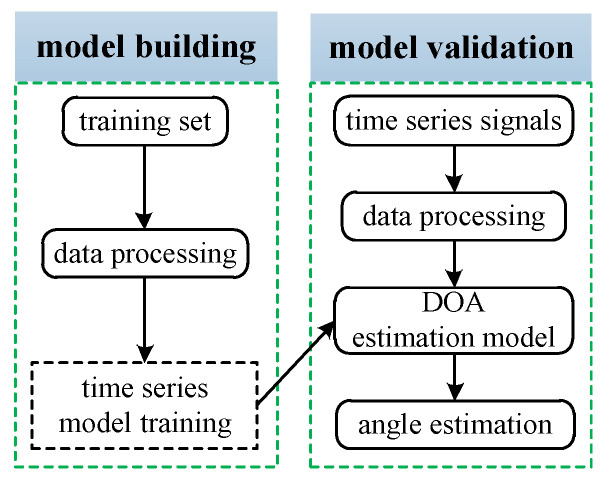
Framework of DOA estimation method based on a neural network with temporal structure.

The output data X(t) of the AVS array has timing information, but the covariance matrix loses the timing structure, which leads to the loss of some features. Therefore, in order to meet the actual needs, this paper proposes to use temporal series data to build two DOA estimation methods based on the neural network for the AVS array. The overall frame is shown in Figure 2. The DOA estimation model with temporal structure neural network mainly includes the DOA estimation model based on LSTM-ATT and DOA estimation model based on Transformer. This section will further elaborate on the two models around the two stages of model construction and model verification.

### 3.1. Model Construction

The model construction mainly completes data processing and model training; the data processing process can be seen in Section 2, and the two model training processes will be introduced in the following sections.

#### 3.1.1. DOA Estimation Model Based on LSTM-ATT

**Figure 3:**
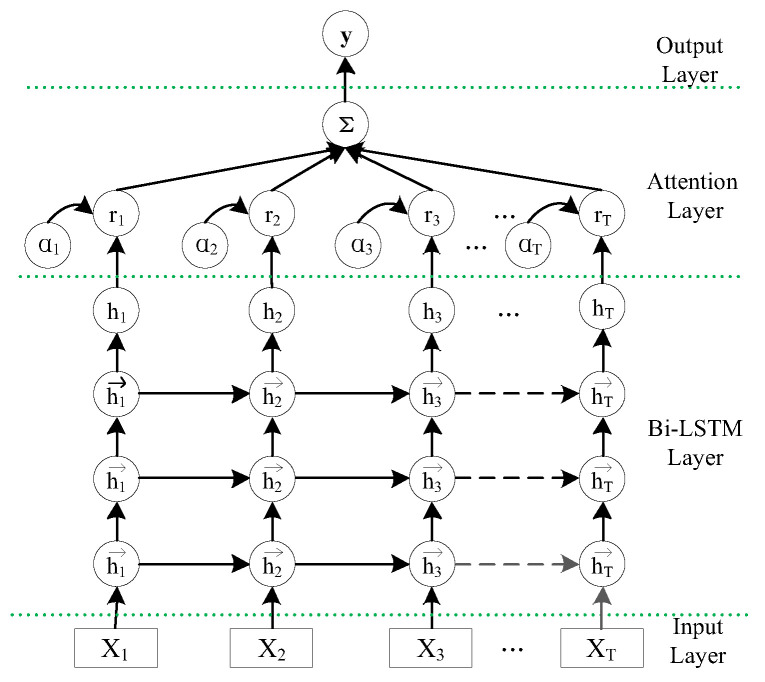
Network structure diagram of the DOA estimation model based on LSTM-ATT.

The DOA estimation model is based on the LSTM network, which can better capture the temporal series information. On this basis, the attention mechanism is added to make the method selectively focus on the more important content of the DOA estimation in the sequence, which can further improve the accuracy of the DOA estimation. As shown in Figure 3, the network structure of the proposed model contains four layers: input layer, LSTM layer, attention layer, and output layer.

**Figure 4:**
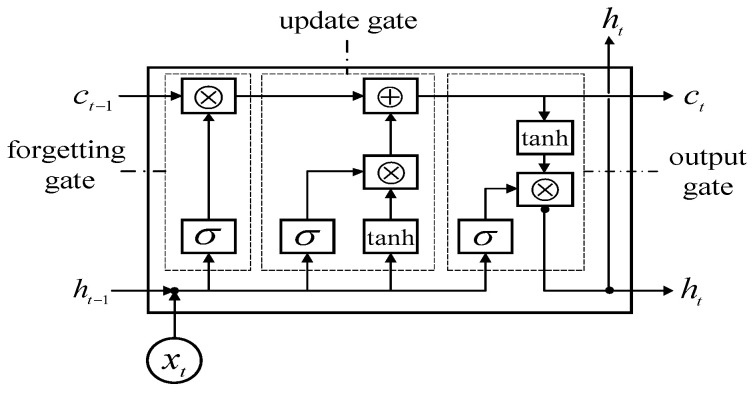
Schematic diagram of the LSTM internal structure.

(1) Input layer: This is used to receive signal data after analysis and processing.

(2) LSTM layer: This completes the extraction of context signals. As shown in Figure 4, the internal structure of the LSTM unit is composed of a forgetting gate $f_{t}$ (included weight parameter $W_{xf},\;W_{hf},\;W_{cf},\;b_{f}$), updating gate $i_{t}$ (included weight parameter $W_{xi},\;W_{hi},\;W_{ci},\;b_{i}$), output gate $o_{t}$ (included weight parameter $W_{xo},\;W_{ho},\;W_{co},\;b_{o}$), and data combination $g_{t}$ (included weight parameter $W_{xc},\;W_{hc},\;W_{cc},\;b_{c}$). We let the unit state, received input, and unit output of the current neuron be $c_{t}$, $x_{t}$, $h_{t}$, respectively, and the cell state and output of the last cell be $c_{t-1}$ and $h_{t-1}$, respectively. The function and mechanism of each door are as follows:

The forgetting gate mainly controls whether to forget the forgetting degree of signal information in the previous unit. The current time-step signal and the hidden state of the previous unit is input, matrix multiplication is performed, and an output in the range of [0, 1] after sigmoid activation is obtained. In the forgetting gate, $f_{t}$ is multiplied by the corresponding elements of the signal of the previous unit so as to control the forgetting degree of the signal information of the previous unit. The closer $f_{t}$ is to 0, the more the information of the previous unit needs to be forgotten; otherwise, the information of the previous unit needs to be retained.

(7) $$f_{t}=\sigma \left(W_{xf}\cdot x_{t}+W_{hf}\cdot h_{t-1}+W_{cf}\cdot c_{t-1}+b_{f}\right)$$

The update gate is used to control the merging of the current time signal input with the signal information of the last unit. It first contains a structure $i_{t}$ similar to forgetting, which is used to select the signal information of the current unit.

(8) $$i_{t}=\sigma \left(W_{xi}\cdot x_{t}+W_{hi}\cdot h_{t-1}+W_{ci}\cdot c_{t-1}+b_{i}\right)$$

Secondly, the signal information of the previous unit and the signal input of the current unit are used to form the signal information $g_{t}$ of the current unit to be selected.

(9) $$g_{t}=\tanh\left(W_{xc}\cdot x_{t}+W_{hc}\cdot h_{t-1}+W_{cc}\cdot c_{t-1}+b_{c}\right)$$

Finally, the signal information $f_{t}$ and $i_{t}$ are used to screen the signal information of the previous unit and the current unit, respectively, then the signal information of the current unit is generated.

(10) $$c_{t}=i_{t}g_{t}+f_{t}c_{t-1}$$

In regards to the output gate, an output $o_{t}$ with a value range in [0, 1] is obtained by sigmoid activation to control the flow of information in the hidden state.

(11) $$o_{t}=\sigma \left(W_{xo}\cdot x_{t}+W_{ho}\cdot h_{t-1}+W_{co}\cdot c_{t-1}+b_{o}\right)$$

(12) $$h_{t}=o_{t}\tanh\left(c_{t}\right)$$

Since the signal data may have large noise, three LSTM layers are used in this paper to extract the data on the upper layers further, respectively.

(3) Attention layer: This layer receives the output of the LSTM layer to generate weights for the signal features at each time step and performs a weighted sum to output the final features. We let the output of the LSTM layer be $H=[h_{1},h_{2},\ldots \ldots ,h_{n}]$, and the attention weight parameter be w. The attention layer generates the final feature through the following process:

(13) M=tanh(H)

(14) $$\alpha =\frac{\exp\left(w^{T}M\right)}{\sum \exp\left(w^{T}M\right)}$$

(15) $$h_{sen}=\tanh\left(H\alpha^{T}\right)$$

(4) Output layer: The output layer has the following structures according to different tasks:

For the probability prediction of incident angle, the output layer consists of a fully connected layer with multiple neurons, the number of which is equal to the number of incident angle categories. This layer receives the features of the attention layer and predicts the probability distribution using softmax output. The model uses an Adam optimizer and multi-class cross-entropy loss. The loss calculation formula is as follows;

(16) $$loss=-\sum_{i=1}^{n}{\hat{p}}_{i}\times \log\left(p_{i}\right),$$

where *p* is the actual label probability distribution, $\hat{p}$ is the predictive label probability distribution, and n is the number of samples.

For direct prediction of incident angle, the output layer consists of a fully connected layer with a neuron. This layer does not use any activation function, it directly outputs the data obtained from the neurons, and the Adam optimizer is adopted to adjust the parameters according to the mean square error loss (MSE). The loss calculation formula is as follows:

(17) $$loss_{\mathrm{MSE}}=\frac{1}{n}\sum_{i=1}^{n}{\left({\hat{y}}_{i}-y_{i}\right)}^{2}$$

where *y* is the true angle of arrival, and $\hat{y}$ is the predicted angle of arrival.

#### 3.1.2. Transformer-Based DOA Estimation Model

The DOA estimation model based on LSTM-ATT in Section 3.1.1 screens the data multiple times, which can retain important signal information and improve the DOA estimation accuracy. However, the disadvantage that the sequence structure can only run in series leads to a long modeling time for LSTM, and a single attention mechanism may run the risk of model overfitting. Therefore, in order to improve the computational efficiency of the DOA estimation model, a DOA estimation model based on the Transformer [22] is established. Transformer abandons the sequence model and no longer inputs the signal according to time steps but as a whole. Moreover, the input signal is divided into multiple segments, which are sent to multiple subspaces, respectively, and then the influence calculation is performed by the self-attention machine in each subspace, and, finally, the attention weight is combined and calculated. Although this method abandons the sequence structure and weakens the connection before and after the time step, the large-scale parallel computing efficiency far exceeds that of the sequence model, which greatly reduces the time required for modeling, and multi-head attention can be regarded as self-attention. The integration not only improves the performance of the attention mechanism but also reduces the risk of model overfitting.

**Figure 5:**
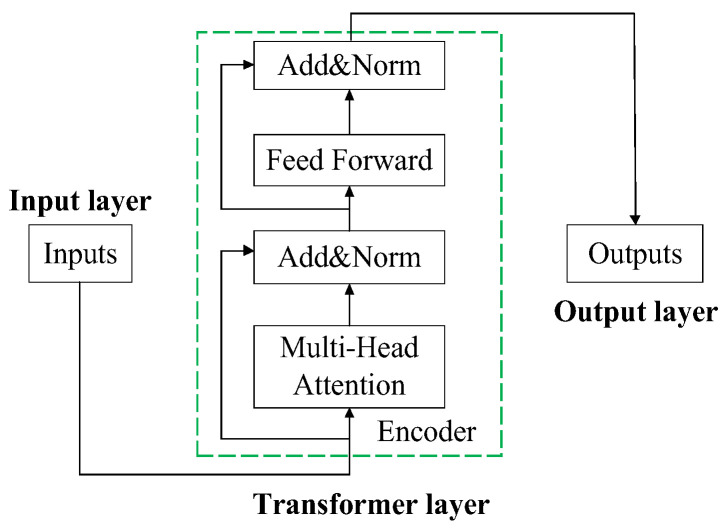
Transformer-based DOA estimation model network structure.

The DOA estimation model based on Transformer is mainly composed of an input layer, a Transformer layer, and an output layer. Its network structure is shown in Figure 5. The sequence signal data is received by the input layer and transmitted to the Transformer for feature extraction to find the DOA in the sequence. More important signals are estimated; the high-level information extracted by the Transformer layer is finally input to the output layer to complete the DOA estimation task.

(1) Input layer: Considering that the input signal is numerically small, adding positional encoding will cause the original signal to be overwritten, so the model used in this paper does not use positional encoding.

(2) Transformer layer: Since the DOA recognition task is relatively single, the Transformer used in this paper does not consider the decoder, but only uses the encoder. After one encoder, the forward conduction formula of the signal context representation obtained by the Transformer layer can be expressed as follows:

(18) $$x_{1}=Norm\left[x+MHA\left(x\right)\right]$$

(19) $$x_{2}=Norm\left[x_{1}+FF\left(x_{1}\right)\right]$$

where, MHA and FF represent the multi-head attention layer and the forward propagation layer, respectively, and Norm represents the layer regularization.

The MHA layer consists of multiple self-attention machines. For a self-attention machine, the forward propagation formula is as follows:

(20) $$Q=w_{Q}x+b_{Q}$$

(21) $$K=w_{K}x+b_{K}$$

(22) $$V=w_{V}x+b_{V}$$

(23) $$Attention\;\left(Q,K,V\right)=\mathrm{softmax}\left(\frac{QK^{T}}{\sqrt{d_{k}}}\right)V$$

*Q*, *K*, *V* is the built-in parameter of the self-attention machine, $w_{Q}$, $w_{K}$, $w_{V}$ is the weight matrix, $b_{Q}$, $b_{K}$, $b_{V}$ is the bias term, $K^{T}$ is the transpose of the matrix *K*, and $d_{k}$ is the length of the signal feature. First, self-attention tries to find the influence of each element in the sequence on other elements. The magnitude of this influence is obtained by applying a vector dot product between two elements, that is, $QK^{T}$ in the formula; secondly, the data is divided by $\sqrt{d_{k}}$ to scale the data so that the gradient of the network is more stable; again, through the softmax function, the influence between elements is converted into a weight vector and finally multiplied by *V* to get the final output. In MHA, several self-attention machines operate independently and in parallel, are respectively responsible for extracting the information of specific positions in the signal, and, finally, perform the same weight calculation in the softmax layer. This structure can be regarded as the result of the integration of multiple self-attentions, which not only improves the signal estimation effect and reduces the possibility of model overfitting, but also improves the computational efficiency and reduces the modeling and prediction time.

The FF layer consists of two fully connected layers. The activation function of the first layer is Relu, and the second layer has no activation function.

The Norm layer regularizes the data to reduce the risk of overfitting, the formula is as follows:

(24) $$Norm(x)=\gamma \times \frac{\left(x-\mu \right)}{\sqrt{\sigma +\varepsilon }}+\beta$$

Among them, μ is the average value of the input, σ is the variance of the input, and ε is a very small disturbance to prevent the variance from being 0; γ and β are the weight parameters of the model, and the initial values are 1 and 0, respectively.

**Figure 6:**
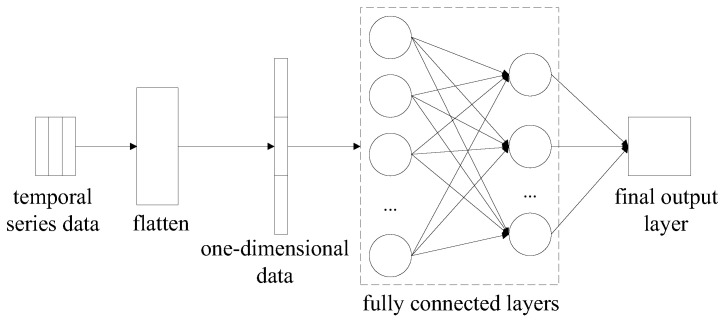
Output layer structure.

(3) Output layer: This consists of a Flatten layer, a fully connected layer, and the final output layer, as shown in Figure 6. The data loses its temporal structure in the Flatten layer and is converted into one dimension in order to complete the angle prediction task; the fully connected layer is used to complete the angle prediction task. Depending on the task, the final output layer can output the probability distribution of the angle or directly output the angle itself.

The model is parameterized using the Adam optimizer according to the multi-class cross-entropy loss (angle probability distribution) or the MSE loss (prediction angle itself). The multi-class cross-entropy loss (angle probability distribution) and the MSE loss calculation formula are shown in the following. The calculation formulas of multi-class cross-entropy loss and MSE loss are shown in Equation (16) and Equation (17), respectively.

### 3.2. Model Validation

For a signal to be predicted that does not participate in training, this paper firstly analyzes and processes the data, inputs the processed data into the two DOA estimation models obtained in Section 3.1, and then completes the probability or direct prediction of the incident angle.

## 4. Simulation Experiment and Verification

### 4.1. Data Set

In order to verify the performance of the proposed DOA estimation method based on LSTM-ATT and Transformer, simulation experiments are conducted for a uniform linear AVS array. The uniform linear array is composed of three AVSs, and the array element spacing is half a wavelength. The incidence angle range is [0°, 180°], one sample is taken every 1°, the number of snapshots is 100, and 1000 samples are taken for each angle. For each incident angle, seven sets of sample data with different SNRs (−15 dB, −10 dB, −5 dB, 0 dB, 5 dB, 10 dB, and 15 dB) were generated. In this paper, taking 1° as the grid density, the problem is likened to a 181-classification problem, that is, 181,000 data are collected as the training data of the neural network under each set of SNR, and the corresponding labels are well marked. The data set is randomly divided into a training set and a verification set according to the ratio of 9:1 for model training and parameter adjustment, and different samples are generated as test sets for verification according to the way that the search angle range is [0°, 180°], and the search interval is 1°. The experiment is conducted 20 times to confirm the results and the average result is reported to avoid randomness.

### 4.2. Parameter Setting

(1) Other parameters of the DOA estimation method based on LSTM-ATT are set as follows:

Adam optimizer is selected;
the learning rate is 1e-4;
and the input batch size is 32.

(2) For the DOA estimation model based on Transformer:

Adam optimizer is selected;
the learning rate is 1e-4;
and the input batch size is 32.

The fully connected layer in the output layer is composed of two dense layers with 512 and 256 neurons, respectively. Relu is used as the activation function.

In order to conduct the simulations, the Pycharm libraries have been used on a computer with Gen Intel(R) core(TM) i7-11700k CPU @ 3.60 GHz and NVIDIA GeForce RTX 3060 GPU.

### 4.3. DOA Estimation Model Performance Analysis

**Figure 7:**
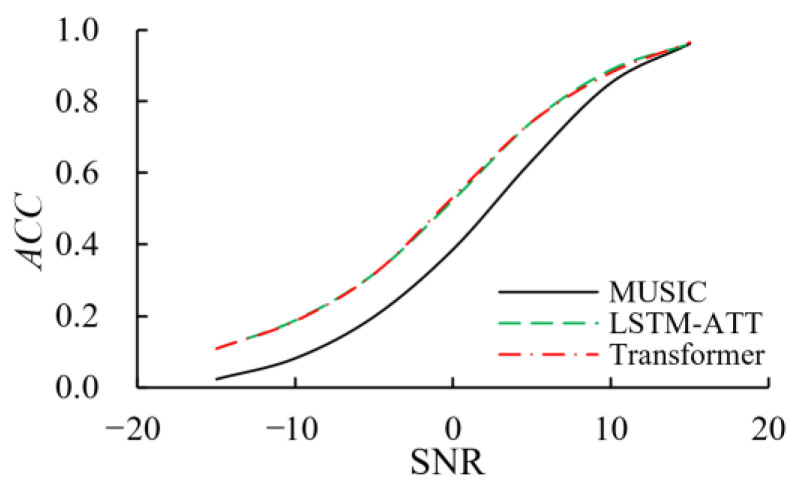
Accuracy of each method under different SNRs.

To analyze the estimation performance of the method proposed in this paper, the accuracy of the DOA estimation methods based on LSTM-ATT and Transformer for AVS array under different signal-to-noise ratios is calculated and compared with the MUSIC method, as shown in Figure 7. In this paper, *ACC* is measured from the perspective of classification, that is, all possible directions are discretization into some fixed angle values. For example, if there is an angle value every 1 degree, these angle values are the classification labels. Under this classification framework, the calculation formula of *ACC* is as follows:

(25) $$ACC=\frac{N_{\mathrm{exact}}}{N_{\mathrm{total}}}$$

In Equation (25), $N_{\mathrm{total}}$ is the total number of tests, and $N_{\mathrm{exact}}$ is the number of correctly classified data points in the test data, that is, the number of correctly classified data points.

It can be seen from Figure 7 that the accuracy of DOA estimation methods based on LSTM-ATT and Transformer are basically the same under different SNRs. With the increase of SNR, the accuracy of each method gradually improves. When the SNR is equal to 15 dB, the accuracy of each algorithm is over 96%. However, when the SNR is −15 dB to 10 dB, the DOA estimation methods based on LSTM-ATT and Transformer both perform better than the MUSIC method. It indicates that the DOA estimation methods based on LSTM-ATT and Transformer not only have a strong anti-noise ability but also can greatly improve the accuracy.

**Figure 8:**
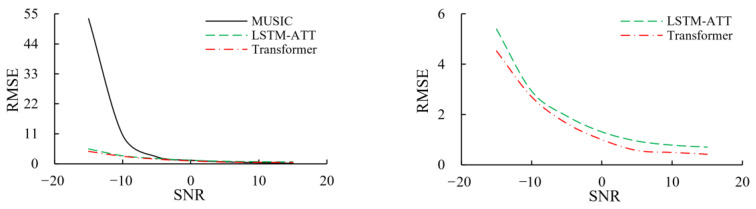
RMSE of each method under different SNR.

Figure 8 is the relationship curve between the root mean square error (RMSE) of the DOA estimation and the SNR of each method. The calculation formula of RMSE is as follows:

(26) $$\mathrm{RMSE}=\sqrt{\frac{1}{n}\sum_{i=1}^{n}{\left({\hat{y}}_{i}-y_{i}\right)}^{2}}$$

It can be analyzed that when the SNR is less than 5 dB, the DOA estimation errors based on LSTM-ATT and Transformer are both significantly smaller than those of MUSIC, and the difference in estimation errors is more obvious with the decrease of the SNR. It shows that the DOA estimation methods based on LSTM-ATT and Transformer have better robustness in harsh environments and also proves the importance of time-series structure in DOA estimation. It can also be seen from the right figure that the DOA estimation error based on Transformer is significantly smaller than that based on LSTM-ATT, indicating that the DOA estimation method based on Transformer has higher prediction accuracy.

### 4.4. Effect of the Incidence Angle on the Performance of the DOA Estimation Method

**Figure 9:**
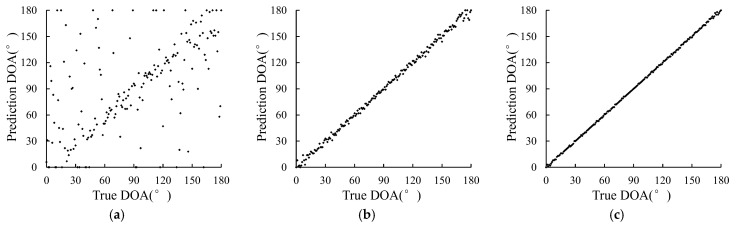
Prediction results of the MUSIC method. (**a**) SNR = −15 dB; (**b**) SNR = −5 dB; (**c**) SNR = 5 dB.

**Figure 10:**
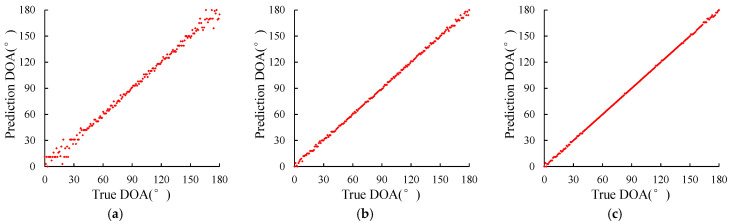
Prediction results of the DOA estimation method based on LSTM-ATT. (**a**) SNR = −15 dB; (**b**) SNR = −5 dB; (**c**) SNR = 5 dB.

**Figure 11:**
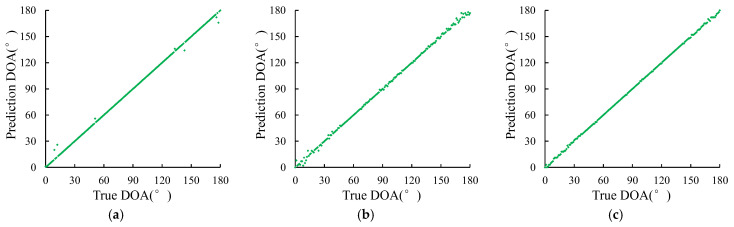
Prediction results of the DOA estimation method based on Transformer. (**a**) SNR = −15 dB; (**b**) SNR = −5 dB; (**c**) SNR = 5 dB.

MUSIC algorithm performs poorly at edge angle. This section mainly studies whether various DOA estimation methods proposed can effectively estimate all angles from 0 to 180 degrees, so as to explore the influence of source incident angle on the method. When the SNR is −15 dB, −5 dB, and 5 dB, a set of signal data with incident angles of 0°–180° are generated, respectively, and then sent to different methods for estimation. The estimation results are shown in Figure 9, Figure 10 and Figure 11.

In Figure 9, the distribution of points at low SNR is very scattered, that is, the estimation accuracy of the MUSIC method at low SNR is very low, and the incidence angle cannot be effectively estimated. With the increase of SNR, the distribution points are gradually concentrated, indicating that the estimation accuracy is gradually increased with the increase of SNR. As can be seen from Figure 10, at each SNR, the distribution of points is more concentrated than that of the MUSIC method, especially when the SNR is −15 dB, indicating that the estimation results of the DOA estimation method based on LSTM-ATT are better than those of the MUSIC method. As can be seen from Figure 11, at SNR −15 dB, the distribution of points is more concentrated than that of the DOA estimation method based on LSTM-ATT. At SNR −5 dB and 5 dB, the distribution of points is comparable to that of the DOA estimation method based on LSTM-ATT, indicating that the estimation result of the DOA estimation method based on Transformer is basically comparable to that of the DOA estimation method based on LSTM-ATT. However, the DOA estimation method based on Transformer has higher estimation accuracy under the condition of low SNR.

### 4.5. Effect of the Number of Array Elements and Snapshots on the Performance of DOA Estimation Method

When SNR is −15 dB, the accuracy of the LSTM-ATT method and the Transformer method is less than 11%, while the accuracy of the MUSIC method is less than 3%. In the environment of small SNR, the most effective method to improve the accuracy of DOA estimation is to increase the number of array elements and snapshot numbers. Therefore, on the basis of Section 4.4, the number of array sources is increased to obtain the accuracy of each method. The accuracy of each method under different array elements and snapshot numbers is shown in Table 1. In Table 1, the accuracy of each method is significantly improved with the increase of array elements at each SNR, especially at low SNR. When the SNR is -15dB, the accuracy of the LSTM-ATT-based method and the Transformer-based method both exceed 30% when *M* = 6, while the accuracy of the MUSIC method is less than 8%. When the SNR is 15dB, the accuracy of each method is more than 99% when *M* = 6. At each SNR, the accuracy of the LSTM-ATT-based method and the Transformer-based method is basically higher than that of the MUSIC method, and the lower the SNR is, the more obvious the advantages of the LSTM-ATT-based method and the Transformer-based method are. When the SNR is less than or equal to 5dB, the accuracy of the LSTM-ATT-based method and the Transformer-based method at *M* = 6 is at least 20% higher than that at *M* = 3. When the SNR is greater than or equal to 10 dB, the accuracy of the LSTM-ATT-based method and the Transformer-based method exceeds 97% when *M* = 6. The impact of the number of snapshots on each DOA estimation method is similar to that of the number of array elements, that is, increasing the number of snapshots will increase the accuracy of each method, and the LSTM-ATT-based method and the Transformer-based method will increase the accuracy more significantly at low SNR. On the whole, the accuracy of the DOA estimation method based on neural networks with temporal structure is significantly higher than that of the MUSIC method, especially under the condition of low SNR. Therefore, under the same number of array elements and snapshots, the temporal characteristics of the signal are of great help to improve the accuracy of DOA estimation.

### 4.6. Time Cost of the Two Estimation Methods Based on Neural Network

DOA estimation of underwater AVS emphasizes timeliness, so the estimation time becomes one of the important criteria to judge the quality of the DOA estimation method. This paper does not compare the proposed methods with the traditional methods because the traditional methods mostly use covariance data, while the method in this paper uses time series data. The time required by each method to run DOA estimation 100 times is counted and recorded. Table 2 shows the computation time and computational complexity of each method. Compared with the DOA estimation method based on LSTM-ATT, the DOA estimation method based on Transformer has a significant reduction in time cost, which is more beneficial to the demand for real-time prediction. In the computational complexity [22], *M* is the number of array elements, *T* is the length of the sequence, and *d* is the input dimension. Transformers can be calculated in parallel, thereby accelerating training speed. LSTM-ATT processes sequences step-by-step, and each time step relies on the calculation results of the previous time step, making it impossible to perform parallel calculations and slower the time. MUSIC involves the calculation of covariance matrices and eigenvalue decomposition, which can be time-consuming with the data.

## 5. Conclusions

In this paper, two DOA estimation methods based on neural network are proposed, both of which are based on the temporal signal data output by the underwater AVS array. One is a DOA estimation method based on LSTM and attention mechanism, which constructs a multi-layer LSTM network to learn the context information of temporal signals and introduces attention mechanism to highlight the features that are important auxiliary to signal estimation so as to improve the estimation accuracy. The other is the DOA estimation method based on Transformer, which divides the timing signal into multiple segments and respectively feeds them into multiple subspaces. Then, the self-attention machine in each subspace calculates the influence, and, finally, the attention weight is combined to improve the calculation efficiency. Through the simulation experiment with a small number of array elements, the following conclusions can be obtained:

(1) Both the DOA estimation method based on LSTM-ATT and the DOA estimation method based on Transformer can effectively predict the direction of the received signal by learning the differences between the received timing signals of different incident directions, and the estimation accuracy is significantly better than that of the MUSIC method, especially when the SNR is low. The advantage is very obvious, which also proves the importance of temporal features for DOA estimation in a real environment.

(2) The accuracy of the DOA estimation method based on Transformer is comparable to that of the DOA estimation method based on LSTM-ATT at each SNR.

(3) The computational efficiency of the vector DOA estimation method based on Transformer is significantly higher than that based on LSTM-ATT.

In the future, the authors plan to combine the output results of the neural network with the output results of the MUSIC method to obtain a more accurate DOA estimation. Specifically, using the output of the neural network as prior knowledge in the MUSIC method allows for a more refined calculation of the signal subspace. However, further research and exploration are needed to fully investigate this.

## Figures and Tables

**Table 1:** Comparison of each method under a different number of array elements and snapshots.

*SNR* /dB	*M* = 3, *K* = 100			*M* = 3, *K* = 500			*M* = 6, *K* = 100		
	MUSIC	LSTM-ATT	Transformer	MUSIC	LSTM-ATT	Transformer	MUSIC	LSTM-ATT	Transformer
−15	0.0233	0.1088	0.1092	0.0623	0.2235	0.2265	0.0714	0.3079	0.3178
−10	0.0817	0.1881	0.1865	0.1902	0.3744	0.3847	0.2616	0.5042	0.5012
−5	0.1987	0.3186	0.3183	0.4218	0.5713	0.5708	0.5271	0.7172	0.7228
0	0.3869	0.5247	0.5330	0.7102	0.7702	0.7835	0.7714	0.8620	0.8608
5	0.6352	0.7439	0.7432	0.9036	0.9121	0.9118	0.9041	0.9324	0.9372
10	0.8525	0.8886	0.8801	0.9823	0.9787	0.9772	0.9672	0.9734	0.9762
15	0.9618	0.9611	0.9643	0.9993	0.9985	0.9828	0.9948	0.9944	0.9930

**Table 2:** Estimation time in different conditions.

DOA Estimation Method	Time/s			Computational Complexity
	*M* = 3, *L* = 100	*M* = 3, *L* = 500	*M* = 6, *L* = 100	
LSTM-ATT	0.0094	0.0334	0.0099	$O(M^{2}\cdot T)$
Transformer	0.0022	0.0032	0.0029	$O(M^{2}\cdot d)$
MUSIC	0.6719	0.6875	6.2969	$O(M^{3})$

## Data Availability

The data presented in this study are available on request from the corresponding author.
